# Big data for big questions: it is time for data analysts to act

**DOI:** 10.4155/fso.15.19

**Published:** 2015-11-01

**Authors:** Pablo Moscato

**Affiliations:** 1 Centre for Bioinformatics, Biomarker Discovery & Information-Based Medicine, The University of Newcastle, Callaghan, New South Wales, Australia

**Keywords:** Alzheimer's disease, big data, cancer, information-based medicine, personalized medicine

## Abstract

**Pablo Moscato speaks to Francesca Lake, Managing Editor** Australian Research Council Future Fellow Prof. Pablo Moscato was born in 1964 in La Plata, Argentina. Obtaining his B.Sc. in Physics at University of La Plata, his PhD was defended at UNICAMP, Brazil. While at the California Institute of Technology Concurrent Computation Program he developed, in collaboration with Michael Norman, the first application of a methodology later called ‘memetic algorithms’, which is now widely used internationally. He is the founding co-director of the Priority Research Centre for Bioinformatics, Biomarker Discovery and Information-based Medicine (CIBM) (2006–present) and the funding director of the Newcastle Bioinformatics Initiative (2002–2006) of The University of Newcastle (Australia). He is also Chief Investigator of the Australian Research Council Centre in Bioinformatics. He is one of Australia's most cited computer scientists. Over the past 7 years, he has introduced a unifying hallmark of cancer progression based on the changes of information theory quantifiers, and developed a novel mathematical model and an associated solution procedure based on combinatorial optimization techniques to identify drug combinations for cancer therapeutics. In addition, he has identified proteomic signatures to predict the clinical symptoms of Alzheimer's disease, among other ‘firsts’. He is a member of the Editorial Board of *Future Science OA*.

## Can you tell us a little about your career to-date, & how you came into bioinformatics?

I had 5 years of undergraduate education in Physics before going to Caltech (CA, USA) in 1988–89. While I was there, I was a Visiting Graduate student and Core Research member of the concurrent computation program. Consequently, I moved to Computational Physics and Computer Science, studying areas of Machine Learning and Artificial Intelligence. My graduate studies took 5 years in total, which I completed at UNICAMP in Brazil.

Overall, my career path has been highly eclectic, starting activities in bioinformatics back in 1993 in models of protein folding. However, it was only when I moved to Australia that I had the right moment and conditions to develop a group that was dedicated full-time to bioinformatics, applied to the biomedical and life sciences.

## Can you tell us a little about what you are working on at the moment?

Our work is basically in large-scale data analytics. We could say that the overall theme is pattern mining and predictive analytics in a variety of areas. Under my direction we have ongoing projects in Alzheimer's Disease; brain, breast and prostate cancer; melanoma; multiple sclerosis and age-related macular degeneration. However, we are also heavily involved in other areas such as computational social sciences. In all cases, we have a particular interest in the individual, so personalized medicine, personalized marketing, personalized services and their relationship with the new digital future are all main themes for us.

## You established the Newcastle Bioinformatics Initiative in 2002. What led to this?

The university needed leadership in the area, and after being hired there was an internal grant scheme for strategic initiatives. I started working for such a bid less than 48 h after my arrival and got my internal project awarded with half a million dollars a few months later. This cemented a project that aimed to break the silos of medical research in different faculties and help the institution to tighten together its strengths in a common goal. The Initiative finished in 2006; one of its objectives was the creation of a Centre, the Centre for Bioinformatics, Biomarker Discovery and Information-Based Medicine (CIBM) so we have more than succeeded in achieving what we promised.

## With CIBM, you have taken models developed through language studies, such as on the works of Shakespeare, & applied them to personalized medicine. How did that come about? Do you think there will be more cross-field developments such as this?

Back in 2005 I discovered that I had a colleague at the university, Prof. Hugh Craig, who was using Principal Component Analysis (PCA) to track variations in the use of English words over decades. He was based on works by Shakespeare and his contemporaries. I proposed to look at his databases and a full collaboration slowly started.

Essentially, like in transcriptomics or proteomics, a literary work, say ‘Hamlet’, is reduced to a ‘spectrum’: the computed observed normalized frequencies of use. As the right way of computing similarities is one of the major themes of our work, I looked at using the Jensen–Shannon divergence to compute the similarities between works. This has led to several papers written about these ideas over the years. We have established the use of the Jensen–Shannon divergence firmly into biomedical research as well as in computational stylistics. For instance, in 2012 a group led by Christopher Burge at MIT published in the journal *Science*, produced an important study in mammals that shows that tissue-specific gene expression programs are largely conserved while alternative splicing is well-conserved in some tissues only. Their results are supported by the use of the Jensen–Shannon divergence and cite our previous work [1]. I believe they could have not done their job right using another type of correlation metric to compute similarities.

A number of unpublished experiments I did with the Jensen–Shannon divergence on works of the English Renaissance Era helped me to forge ideas about how to use it in cancer and neurodegeneration. I worked from 2007 to 2010 on the theme of detecting biomarkers of use based on covariations of gene expression with information theory-quantifiers in cancer and Alzheimer's Disease. I submitted my findings within 24 h of difference in two articles to PLoS ONE in 2009, which were both accepted in 2010. We basically propose a new paradigm here for biomarker discovery, understanding gene expression as ‘a message’ and tracking progressions of disease by monitoring the changes in its information content. I dare to say, it is a new paradigm that was applied to two widely different scenarios. A unifying hallmark for cancer and neurodegeneration [2,3].

With this methodology, I pointed to a particular protein in Alzheimer's Disease, VSNL1 (see Fig. 19 of [3]) as a particularly interesting one to track progression. I also presented a poster on the subject at the International Conference of the Alzheimer's Disease association in Hawaii in 2010. Today, results in CSF are now confirming that the presence of VSNL1 is correlated with disease progression [4–6]. It has been very satisfying for me to see how relatively simple methods from mathematics and Information Theory, using a public domain dataset with only a few samples, can help to point out particular biomarkers of true value.

I am convinced that this is indeed a right approach, to build bioinformatics capacity from a true interdisciplinary Data Science capacity. As a consequence, we conduct studies in other areas of computational social science, machine learning, etc., with all the projects mutually informing each other and finally contributing to biomedical research.

## You are co-leader of Hunter Medical Research Institute's (HMRI) Information-based Medicine Program; what does this role entail?

The HMRI is one of the largest institutes of its type in the country. It is organized in seven different research programs. Our program is in some sense ‘horizontal’ as our quest for new methodologies for biomarker identification for personalized medicine and detection tests relates with the activities of all other programs. As co-director of the program I direct activities that complement and innovate in medical research by introducing novel analytical procedures and a ‘Big Data’ approach to understand disease progression. As a consequence we have activities in several diseases and we are not limited in scope. This freedom gives a lot of energy to our group.

## You've made a number of significant advances in human health through your research. What did you find the most rewarding, & why?

What an interesting question! We do not generally stop to think about it. I guess that there are so many open problems that closing some of them gives already a big reward and opens more challenges ahead, so that keeps us always motivated. Let's see, in the area of Alzheimer's disease, through the analysis of different datasets, we are giving a clear hope that we can predict the disease several years before clinical symptoms manifest. In the area of cancer, we have shown that the use of Information Theory can guide the selection of biomarkers which allow us to track the progression of the disease. Overall, we are showing the power of other areas of applied mathematics and computer science, expanding the horizon of what statistical methods can bring for data analysis in a major way which is particularly rewarding for me.

## What do you think the challenges are for data scientists in medical research?

Data scientists, after years of collaborative endeavors with medical and life-science researchers, are ready. I hope they should really step up and be the chief leader of their own projects in medical research. I think that there are very talented data scientists working in biomedicine who have the experience to make great advances if they are the drivers of their own research. For society, they will have a major role in designing a more efficient medical research enterprise. For this to happen, they also need to know the biomedical aspects well and work collaboratively for many years, but eventually it would require them to take courage and design their own experiments. Ideally, data-rich scenarios should be designed for large projects delivering multiple objectives. It is a major Systems Engineering redesign of medical research via embedding a data-driven culture in the health sector.

## How do you think we should go about addressing these?

The funding institutions of medical research should be establishing a percentage of the grants to be directed to projects in which the lead chief investigator should be a data scientist. The projects should be judged by a panel of specialists. A couple of biostatisticians in a panel is something of the past, I mean a multidisciplinary group with varied expertise in computer science, applied mathematics, software and systems engineering, etc. With significant support of these highly interdisciplinary projects we will have a new perspective. I dare to say that part of the current problem with medical research is due to the analytics being regarded as ‘a service’. We need a major revision of the national and international funding schemes and how interdisciplinarity should be supported and encouraged, but also be promoted with a truly multidisciplinary leadership.

## How do you see medicine changing as we continue to embrace ‘big data’?

The future always brings a mixed composition. The television did not kill the radio; the cinema did not kill the theatre. Equally, Big Data is not here to kill clinical trials, but it is here to stay. The revolution that Computer Science brings in terms of hypothesis generation, powered by a data-driven approach to scientific exploration, will shake everything. The winners in this revolution will be those that will use it as a tool for reducing costs and use the savings for innovation. How many studies around the world use a great amount of funds to define controls of the study? We are dilapidating resources by a lack of political will to organize medical research as other sciences have organized for subatomic particle physics. We need a change. I agree with Leroy Hood when he said in 2010 “*Medicine is going to become an information science*” [7]. I am glad to say that we called ‘Information-based Medicine’ at our Center 4 years before, pointing the path to this inevitable conclusion.

## Finally, if you had unlimited resources available, what research would you perform & why?

I would need an infinite amount of paper to answer this one! But let me see again… this is a tricky question as if I am in a job interview! Let then me say ‘I will’ instead of ‘I would’ to deliver my promise.

Unlimited resources are not going to give you unlimited numbers of colleagues to work with you, so I would invest in changing the hearts and minds of the best researchers and strengthen the discovery and quantitative aspects of medical research.

I will also heavily invest in expanding the number of academics (and their salaries!) as well as research fellows working in universities. I will do this by aiming at maximizing the diversity of skills in each institution, creating mechanisms for intramural and extramural collaboration.

I will reformulate the pharma, which I think has significant resources yet struggles to have the change in paradigms that would make it more efficient. I feel that the reduction on the number of companies in the area during the past decades is a clear sign that we are in serious trouble due to lack of capacity for innovation. I would put pharma to work cooperatively with the academia in areas where we will preserve free inquiry.

I will fund high-gain/high-risk projects in areas like network pharmacology, drug repositioning, combinatorial therapeutics, etc., as well as in analytical methods in computer science, artificial intelligence and cognitive computing. I will support projects that allow the Healthcare sector to ‘Learn from Data’ as part of a continuous improvement reformulation of their operations. I will also develop global Open Data initiatives.

I will work toward an integrated global-scale system for Electronic Health Records and data management and ethical approvals. I think that we should give more control to the individuals to guide their decisions regarding to their health and the ownership of their data. I would then modify curricula in primary and secondary schools to prepare individuals to manage their health, a life-long activity that they could be helping to monitor with novel technologies. We could then aim for each individual to have the same level of care that a jetliner now has, with constant monitoring of its health. Consequently, I will also massively invest in more accurate and less invasive early-detection tests that could help to detect disease at an early stage.

I will also put funds into solving the ‘big questions’ instead of the ‘next step research’.

But, in some sense I was tricking you; since I have unlimited amount of funds, I can allocate unlimited amounts of funds to all these initiatives, and still have unlimited amounts. I do not need to care about percentages of finite resources. However, the battle for the hearts and minds of our researchers to work on a serious paradigm change remains, we need a cultural change [7]. We would not have 21st century medicine with 19th century ideas of how to do medical research and scientific discovery. The time to act is now.
